# Development of a Quantitative Assay for the Characterization of Human Collectin-11 (CL-11, CL-K1)

**DOI:** 10.3389/fimmu.2018.02238

**Published:** 2018-09-28

**Authors:** Rafael Bayarri-Olmos, Nikolaj Kirketerp-Moller, Laura Pérez-Alós, Karsten Skjodt, Mikkel-Ole Skjoedt, Peter Garred

**Affiliations:** ^1^Laboratory of Molecular Medicine, Department of Clinical Immunology, Faculty of Health and Medical Sciences, Rigshospitalet, University of Copenhagen, Copenhagen, Denmark; ^2^Department of Cancer and Inflammation Research, University of Southern Denmark, Odense, Denmark

**Keywords:** CL-11, collectin, complement system, lectin pathway, zymosan

## Abstract

Collectin-11 (CL-11) is a pattern recognition molecule of the lectin pathway of complement with diverse functions spanning from host defense to embryonic development. CL-11 is found in the circulation in heterocomplexes with the homologous collectin-10 (CL-10). Abnormal CL-11 plasma levels are associated with the presence of disseminated intravascular coagulation, urinary schistosomiasis, and congenital disorders. Although there has been a marked development in the characterization of CL-11 there is still a scarcity of clinical tools for its analysis. Thus, we generated monoclonal antibodies and developed a quantitative ELISA to measure CL-11 in the circulation. The antibodies were screened against recombinant CL-11 and validated by ELISA and immunoprecipitation of serum and plasma. The best candidates were pairwise compared to develop a quantitative ELISA. The assay was validated regarding its sensitivity, reproducibility, and dilution linearity, demonstrating a satisfactory variability over a working range of 0.29–18.75 ng/ml. The mean plasma concentration of CL-11 in healthy controls was determined to be 289.4 ng/ml (range 143.2–459.4 ng/ml), highly correlated to the levels of CL/10/11 complexes (*r* = 0.729). Plasma CL-11 and CL-10/11 co-migrated in size exclusion chromatography as two major complexes of ~400 and >600 kDa. Furthermore, we observed a significant decrease at admission in CL-11 plasma levels in patients admitted to intensive care with systemic inflammatory response syndrome. By using the in-house antibodies and recombinant CL-11, we found that CL-11 can bind to zymosan independently of calcium by a separate site from the carbohydrate-binding region. Finally, we showed that CL-11/MASP-2 complexes trigger C4b deposition on zymosan. In conclusion, we have developed a specific and sensitive ELISA to investigate the ever-expanding roles of CL-11 in health and disease and shown a novel interaction between CL-11 and zymosan.

## Introduction

CL-11 (alias collectin-11 and CL-K1) is a pattern recognition molecule (PRM) of the lectin pathway of complement with diverse functions such as pathogen recognition, tissue homeostasis, and embryonic development (1–4). It is the most recently discovered member of the collectin family, mammalian C-type lectins involved in the innate immune response of the host. Collectins recognize conserved pathogen-specific structures and altered-self molecules and mediate their removal by, among others, agglutination, complement activation, and modulation of inflammatory and adaptive immune responses (5). Collectins share a common bouquet-like structure, with monomeric subunits that assemble into oligomers of trimers. Each monomeric subunit is, in turn, composed of a cysteine-rich N-terminal region, a collagen-like domain, an α-helical neck domain, and a C-terminal carbohydrate recognition domain (CRD). The biological functions of the collectins rely on the high-avidity binding resulting from the oligomerization and clustering of the CRDs.

CL-11 is a secreted collectin found in the circulation (6–8). Elevated CL-11 plasma levels are associated with a decreased risk of contracting urinary schistosomiasis (9) and the presence of disseminated intravascular coagulation (8), characterized by simultaneous clotting and bleeding that frequently leads to multiple organ failure and death. CL-11 deficiency is one of the causes of the 3MC (Malpuech, Michels, Mingarelli, and Carnevale) syndrome (3, 10), a congenital disorder associated with craniofacial dysmorphism, mental retardation, growth deficiency, and physical abnormalities. CL-11 is expressed ubiquitously in the body and it appears to play an important role in maintaining homeostasis in the tissue of expression (1, 11). During embryonic development, CL-11 is highly expressed in the craniofacial cartilage and vertebral bodies, where together with MASP-3 and CL-10, may act as a guidance cue for neural crest cell migration (12). In the mature kidney, locally-produced CL-11 appears to be critical for the development of renal injury after hypoxic and hypothermic stress (13), while in the retina local CL-11 facilitates phagocytosis of photoreceptor outer segments and modulates cytokine production in the retina (4).

Recently, CL-11 has been observed to circulate in complex with another member of the collectin family, CL-10 (collectin-10, CL-L1), forming high molecular weight heterocomplexes (i.e., CL-10/11) that interact strongly with the MASPs (14). Upon binding of CL-11 or CL-10/11 heterocomplexes to carbohydrate structures on the surface of microorganisms, the MASPs activate complement by cleaving C4 and C2 and generating the lectin pathway C3 convertase (C4b2a) (2, 15). Besides their role as the central lectin pathway enzymes, the MASPs are also involved in a variety of biological processes (16–18). MASP-1 has a particularly broad substrate-specificity and it is known to participate in the coagulation cascade, inflammatory reactions, and cell activation; MASP-2 is associated with the development of renal ischemia/reperfusion injury; while MASP-3 might be involved in the modulation of the alternative pathway by activating pro-FD in resting blood.

The multiplicity of biological functions attributed to CL-11 results from its ability to both associate with the MASPs and to interact with a broad range of self and non-self structures, such as yeast, bacteria, viruses, and apoptotic cells (1, 6, 19). Glycan array studies revealed that CL-11 has a preference toward high-mannose oligosaccharides and a subset of fucosylated glycans (10). This unusual binding specificity, different from the other collectins, can be explained by the presence of an extended binding site on the CRD that recognizes both the terminal and the penultimate sugar. Moreover, CL-11 has been observed to bind to certain ligands—e.g., nucleic acids (19), Aspergillus (2), and 9-O-acetylneuraminic acid (10)—in the absence of calcium via a binding site distinct from the carbohydrate-binding region.

Though the biological importance of CL-11 is evident, there is still a scarcity of clinical tools for its analysis. In this study, we generated monoclonal antibodies (mAbs) for the study of human CL-11. The mAbs were screened against serum and plasma using different immunological methods. Furthermore, we developed a quantitative ELISA based on two mAbs to measure the concentration of CL-11 in the circulation. The assay was thoroughly validated and used to determine the levels of CL-11 in plasma from healthy individuals. Our measurements of CL-11 are in good agreement with the literature and are tightly associated with those of CL-10/11. By size exclusion chromatography of plasma, we show that CL-11 forms complexes of 300–400 kDa and >600 kDa with a distinct composition of CL-10 glycosylation states and CL-11 isoforms. Finally, we demonstrate that CL-11 recognizes zymosan in a calcium-independent manner, and triggers C4b deposition in the presence of MASP-2.

## Materials and methods

### Buffers

The following buffers were used: PBS (10.1 mM disodium phosphate, 1.5 mM monopotassium phosphate, 137 mM NaCl, 2.7 mM KCl), PBS-Tw (PBS, 0.5% Tween-20), TBS-Tw ± Ca/EDTA/Mg-EGTA (10 mM Tris, 150 mM NaCl, 0.05% Tween-20, ±2.5 mM CaCl_2_/10 mM EDTA/2 mM MgCl_2_, 10 mM EGTA), sample buffer (TBS-Tw-EDTA, 0.1% v/v bovine serum), barbital-Tw ± EDTA (4 mM sodium barbitone, 145 mM NaCl, 2.6 mM CaCl_2_, 2.1 mM MgCl_2_, with 0.05% Tween-20, ± 5 mM EDTA).

### Generation of Anti-CL-11 monoclonal antibodies (mAbs)

Two outbred NMRI mice were immunized subcutaneously three times with 25 μg of purified recombinant CL-11 (rCL-11) (R&D, USA) adsorbed to Al(OH)_3_ and diluted 1:1 with Freund's incomplete adjuvant (Sigma-Aldrich, USA). Three days prior to the fusion, the mice received a final intravenous injection of 25 μg of antigen and adrenalin. The fusion and subsequent selection were done according to the principles of the hybridoma technology (20). Positive clones were screened in MaxiSorp microtiter plates (Thermo Fisher Scientific, USA) coated with 0.6 μg/ml of polyclonal antibody (pAb) rabbit anti-CL-11 (15269-1-AP, Proteintech, USA) and in-house rCL-11 as antigen. Selected mAbs were purified using a HiTrap Protein G column in an Äkta Pure system (both from GE Healthcare, UK). Briefly, clarified hybridoma supernatant was applied onto the column and washed until a stable UV reading was observed. Bound antibodies were eluted with 0.5% citric acid and neutralized with 0.1 M Tris pH 9. Elution fractions were pooled and dialyzed against PBS. MAbs were biotinylated with 20% w/w (+)-Biotin N-hydroxysuccinimide ester (Sigma, USA) for 3 h at room temperature (RT), followed by dialysis against PBS to remove unreacted biotin.

### Production of recombinant proteins

Recombinant CL-11 (rCL-11) and CL-10 (rCL-10) were produced in Flp-In^TM^-CHO cells (Invitrogen, USA). The coding sequences (NM_024027.4 and NM_006438.4, respectively) were retrieved from the RefSeq NCBI nucleotide database and cloned into pcDNA^TM^5/FRT vectors flanked by two Flp recombinase target (FRT) sites. Stable transfectants were generated by co-transfecting the coding vectors with a pOG44 plasmid encoding the Flp recombinase. Positive clones were selected for Hygromycin resistance and grown in Ham's F-12 Nutrient Mix supplemented with 0.5% FBS, 0.1 mg/ml of Penicillin/Streptomycin (Sigma) and 2 mM of L-Glutamine (all from Gibco, Thermo Fisher Scientific unless otherwise stated) according to the manufacturer's recommendations. The concentration of rCL-11 in the supernatant was calculated using commercially-available rCL-11 (R&D) as calibrator.

### Immunoprecipitation

Anti-CL-11 mAbs Hyb-15 and Hyb-17 (described in section Generation of Anti-CL-11 Monoclonal Antibodies and Development of a CL-11 Specific Sandwich ELISA), or mouse IgG1κ isotype control (BD Biosciences, USA) (2 μg each) were coupled to 25 μl of sheep anti-mouse IgG Dynabeads (Invitrogen) end-over-end for 30 min at RT. The conjugated beads were washed with PBS-Tw and incubated end-over-end 1 h at RT with serum diluted 1:1 in TBS-Tw-EDTA. After washing with PBS-Tw, bound CL-11 was eluted with 0.5% citric acid and subjected to SDS-PAGE as described below.

### SDS-Page and western blot

Denatured samples were separated by electrophoresis in NuPAGE® Tris-Acetate 3–8% gels (Invitrogen, USA) according to the manufacturer's recommendation. Proteins were blotted onto a PVDF membrane (Amersham Bioscience, UK). Membranes were blocked with 5% skim milk (Merck Millipore, USA) and incubated overnight with 0.2 μg/ml of pAb rabbit anti-CL-11 (15269-1-AP, Proteintech) followed by 1 h incubation with pAb swine anti-rabbit-HRP (P0399, Dako, Denmark) in a 1:5000 dilution. After thorough washing, the membranes were developed with SuperSignal™ West Femto Maximum Sensitivity Substrate (Thermo Fisher Scientific) and imaged with Microchemi (Bio-imaging systems, Israel).

### CL-11 specific sandwich ELISA

MaxiSorp microtiter plates were coated overnight at 4°C with 2 μg/ml mAb Hyb-17 in PBS. Serial dilutions of the calibrator, serum and plasma samples in sample buffer were applied to the plate and incubated for 2 h, followed a 2 h incubation with 2 μg/ml of biotinylated mAb Hyb-15 in PBS-Tw. Streptavidin-HRP conjugate (RPN1231V, Sigma) was added to the wells for 1 h in a 1:2000 dilution in PBS-Tw. TMB One (KemEnTec Diagnostics, Denmark) was used as substrate. The reaction was stopped with 0.2 M H_2_SO_4_ and the optical density (OD) was recorded at 450 nm using an ELx80 absorbance reader (BioTek, USA). Plates were washed with PBS-Tw between steps and unless otherwise stated all incubations took place at RT. The optimal combination and concentration of mAbs was determined using a two-dimensional of capture and detection antibodies against serial dilutions of serum and plasma and rated according to their signal intensity and signal-to-noise ratio. The optimal incubation times and sample buffer composition were determined based on the same criteria.

#### Assay validation: parallelism, precision, limit of detection, working range, and variation

Supernatant from Flp-In CHO cells expressing rCL-11 was used as calibrator. The calibrator was applied in a two-fold dilution to generate an eight-point curve with a concentration ranging from 37.5 to 0.29 ng/ml. OD values from serial dilutions of the calibrator, commercial rCL-11 (R&D), serum and EDTA plasma pools (25 and 10 donors, respectively) were logistically-transformed and a log(agonist) vs. response equation was applied to evaluate the parallelism between the best-fit hill slopes. The calibrator was stored at −20°C in single-use aliquots. The limit of detection of the assay was expressed as the background absorbance plus two times the standard deviation (SD). The precision of the assay was evaluated by calculating the ratio between the OD and the mean OD of the triplicates of five donors over a 10-point dilution curve. The dilution linearity was calculated as the ratio between the interpolated concentration for each dilution and the mean concentration calculated from the linear part of the curve. The working range was determined as the concentrations for which both the ratio OD/mean OD and ratio concentration/mean concentration had a coefficient of variation (CV) <20%. Intra-assay variation was calculated as the CV of a plasma pool in 40 wells of a single microtiter plate. To calculate the inter-assay variation, serum and plasma pools were run in triplicates on four separate plates over six different days.

#### Stability of CL-11 in serum and plasma samples

Serum and plasma samples from three healthy individuals were stored at 4°C, RT, or 37°C for up to 72 h. To study the effect of repeated freeze-thaw cycles, serum and plasma samples from a healthy donor were frozen at −80°C and thawed at RT five times. The levels of CL-11 were compared to a control sample subjected to a single freeze-thaw cycle.

### Measurement of CL-10/11 complexes in plasma

MaxiSorp microtiter plates were coated overnight at 4°C with 2 μg/ml mAb anti-CL-10 (HM2356, Hycult) in PBS. Serial dilutions of the calibrator and EDTA plasma 1:40 in sample buffer were incubated for 2 h. Detection and development were performed as described in section CL-11 Specific Sandwich ELISA.

### Serum and plasma samples

The concentration of CL-11 and CL-10/11 complexes in the circulation was determined from blood of Danish healthy volunteers (*n* = 126). In addition, CL-11 levels were measured in plasma from patients at admission to an Intensive Care Unit at Rigshospitalet diagnosed with systemic inflammatory syndrome (*n* = 65). The patients have been described elsewhere (21). Informed consent was obtained from the patients or their relatives in accordance with the Helsinki Protocol. The study was approved by the local ethics committee for Copenhagen County (record no. KA 96097). Serum and plasma were processed within 1 h of blood collection and stored at −80°C. Plasma was obtained by incubating fresh blood with EDTA, citrate, or hirudin—a naturally occurring anticoagulant that binds to and inhibits activated thrombin while maintaining complement activity in blood.

### Size exclusion chromatography

Serum (100 μl) from a pool of blood donors was applied into a Superdex 200 HR 10/30 column (GE Healthcare) at a flow rate of 0.5 ml/min at RT with PBS 2 mM EDTA. The relative molecular weight of CL-11 and CL-10/11 complexes was estimated using a gel filtration marker kit (29–700 kDa, Sigma). CL-11 and CL-10/CL-11 levels in the elution fractions were measured by ELISA as described previously. Results were confirmed by western blotting following Trichloroacetic acid (TCA, Sigma) precipitation. Briefly, elution fractions (500 μl) were incubated 30 min on ice with 10% v/v TCA and centrifuged at 13,000 × g for 30 min at 4°C. Supernatants were discarded and the pellet was washed with ice-cold acetone (Sigma) at 13,000 × g for another 30 min at 4°C. Dry precipitates were resuspended in SDS loading buffer and separated by SDS-PAGE as described above. Anti-CL-10 (0.05 μg/ml, HM2356, Hycult) and pAb rabbit anti-CL-11 (0.05 μg/ml, 15269-1-AP, Proteintech) were used as detection antibodies with rCL-10 and rCL-11 (in-house) as controls.

### Binding of rCL-11 to ligands

Ligand interaction studies were performed in the fluid and solid phase. Unless otherwise stated, all reagents were purchased from Sigma. Fluid phase binding was analyzed by incubating 100 μl of zymosan particles (100 μg, Z4250), mannan-agarose (M9917), D-mannose-agarose (M6400), and Sepharose CL-2B (CL2B300) with 2.75 μg of rCL-11 in 500 μl of TBS-Tw-Ca or TBS-Tw-EDTA for 1.5 h at RT in an end-over-end shaker. Samples were washed thrice with their respective buffer and centrifuged at 3000 × g for 30 min. Pellets were resuspended in SDS loading buffer and subjected to SDS-PAGE using anti-CL-11 Hyb-6 (2 μg/ml) and pAb anti-CL-11 (15269-1-AP, Proteintech) as detection antibodies under non-reducing and reducing conditions, respectively.

Solid-phase binding was determined on microtiter plates coated with zymosan (Z4250), mannan (M7504), BSA (A2153), and D-mannose-BSA (NGP1108, Dextra, UK) in two-fold dilutions starting at 10 μg/ml in PBS overnight at 4°C. The plates washed between steps with TBS-Tw, and unless otherwise stated all incubations took place at RT. rCL-11 (1 μg/ml) was diluted in TBS-Tw-Ca, TBS-Tw-EDTA or TBS-Tw-Mg-EGTA and incubated for 2 h. Hyb-15 (2 μg/ml) in matching buffer was used as detection antibody for 2 h, followed by 1 h incubation with Streptavidin-HRP conjugate (RPN1231V) in a 1:2000 dilution. Development was performed as described previously.

To study the effect of soluble inhibitors on the binding to zymosan and mannan, rCL-11 was incubated with two-fold dilutions of L-fucose (F2252), N-acetyl-glucosamine (A3286), or human genomic DNA (isolated in-house) in TBS-Tw-Ca or TBS-Tw-EDTA for 1 h. Protein/ligand mixes were applied to Maxisorp plates pre-coated with zymosan or mannan (10 μg/ml) and were allowed to bind for 2 h. Quantification of bound rCL-11 was carried out as described before.

### CL-11 mediated c4 deposition on zymosan

Microtiter plates were coated as for the solid-phase binding studies. rCL-11 (2 μg/ml) in barbital-Tw or barbital-Tw-EDTA was applied to the wells and incubated for 2 h at RT, followed by rMASP-2 produced in CHO DG44 cells (0.5 μg/ml, in-house) in barbital-Tw or barbital-Tw-EDTA for 4 h at 37°C. Subsequently, purified C4 (0.5 μg/ml, CompTech, USA) in barbital-Tw was added to the plate for 1 h 37°C. Polyclonal rabbit anti-C4c (2 μg/ml, Dako) was used as a detection antibody for 1.5 h at RT, followed by swine anti-rabbit-HRP (0.15 μg/ml, P0399, Dako). The plates were washed between steps with barbital-Tw. Development and data recoding were carried out as before.

### Statistical analyses

Statistical analyses were performed using GraphPad Prism version 7.02 (GraphPad Software, USA). Measurements of samples and the calibrator were performed in duplicates unless otherwise stated. The concentration of CL-11 in serum and plasma was interpolated by regression analysis using a four-parameter logistic curve fitting. Effect of storage of CL-11 concentration and differences between measured CL-11 in serum and plasma samples was analyzed using two-way ANOVA with Dunnett's multiple comparisons tests. Two-tailed person correlation coefficient was used to determine the correlation between CL-11 and CL-10/CL-11 levels. Differences between CL-11 levels in controls and SIRS patients were analyzed with the Mann-Whitney test. The half maximal inhibitory concentration (IC50) was calculated using the inhibitor concentration vs. response equation on a nonlinear curve fitting constraining the top and bottom parameter to equal 100 and 0, respectively. The significance of observed differences on C4 deposition on zymosan was assessed via one-way ANOVA with Tukey's corrections for multiple comparisons. Data are represented as mean ± SEM of three independent experiments.

## Results

### Generation of Anti-CL-11 monoclonal antibodies and development of A CL-11 specific sandwich ELISA

**Figure 1:**
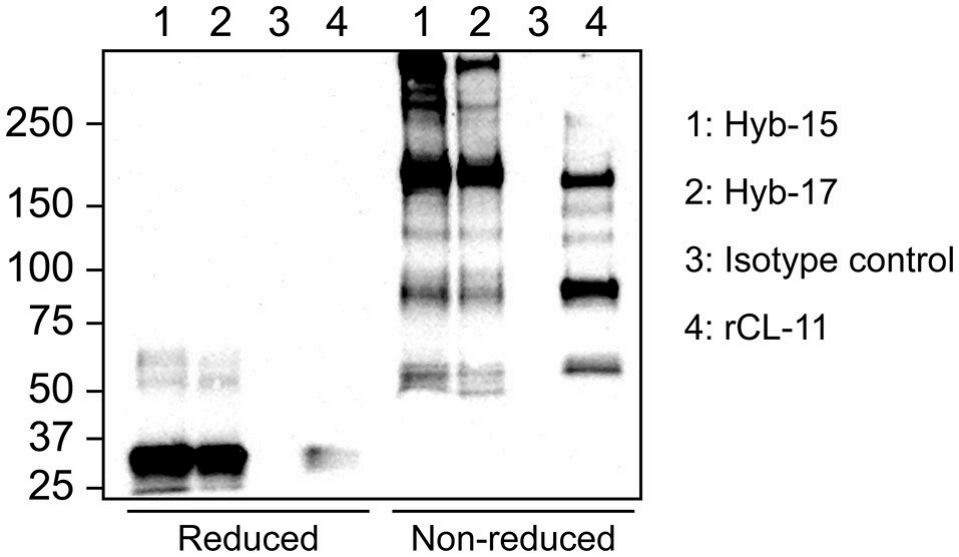
Western blot of serum after immunoprecipitation using CL-11 specific mAbs Hyb-17 and Hyb-15 or a mouse IgG1κ isotype control antibody under reducing and non-reducing conditions. Supernatant from CHO cells expressing rCL-11 was used as positive control. The blots were developed with pAb rabbit anti-CL-11.

Stable monoclonal hybridoma populations were generated by several rounds of cloning by limiting dilution. Each cloning round, culture supernatants were screened by indirect ELISA with rCL-11. Selected positive clones were characterized further by immunoprecipitation of native CL-11 from serum followed by western blotting. As seen in Figure 1, under reducing conditions both mAbs Hyb-15 and Hyb-17 precipitated a protein with an apparent molecular mass of ~34 kDa corresponding to the CL-11 monomer. Under non-reducing conditions, a characteristic ladder-like banding pattern is visible with a major protein band around 180 kDa and several others larger than 250 kDa indicating that CL-11 assembles into high molecular weight oligomers held together by interchain disulfide bridges. rCL-11 used as positive control appeared to form predominantly lower oligomeric forms. An equivalent banding pattern was also seen with the other tested mAbs (data not shown). No bands were apparent when using a mouse IgG1κ control as precipitating antibody.

The best candidates were pairwise screened by sandwich ELISA against a serum and plasma pool. The optimal combination and concentration of mAbs was determined using a two-dimensional of capture and detection antibodies and rated according to their signal intensity and signal-to-noise ratio. Based on their properties, Hyb-17 and Hyb-15 were selected as capture and detection antibody, respectively. No signals were obtained when an irrelevant mouse antibody was used as capture or detection antibody (data not shown).

### Assay validation

**Figure 2:**
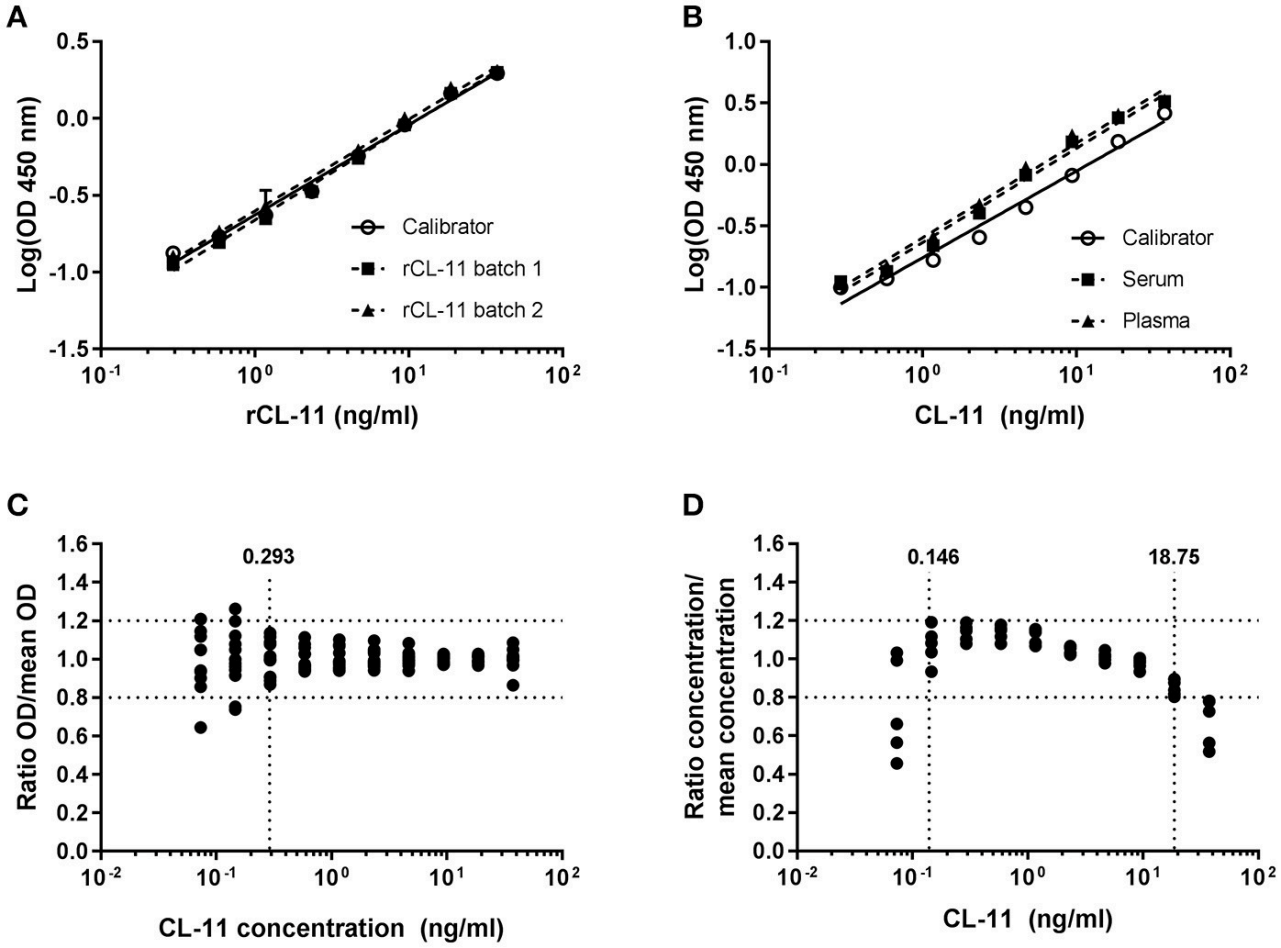
Sandwich ELISA validation. Parallelism between serial dilutions of rCL-11 produced in CHO cells, used as calibrator, and two different batches of purified rCL-11 **(A)** and a serum and plasma pool **(B)** over a 10-point serial dilution series using a log(agonist) vs. response equation (*R*^2^ > 0.99 for all curves). Error bars indicate the standard deviation of the mean of duplicate measurements. **(C)** Precision of the assay on plasma from five donors calculated as the range of concentrations for which the variation of the ratio OD/mean OD of the triplicates is < 20% (i.e., > 0.293 ng/ml). **(D)** Dilution linearity on plasma from five donors calculated as the range of concentrations for which the variation of the interpolated concentration/mean concentration of the triplicates is < 20% (i.e., 0.146–18.75 ng/ml).

Supernatant from CHO cells expressing rCL-11 was used as calibrator. A four-parameter fit model using the equation log(agonist) vs. response was applied to estimate the concentration of rCL-11 in the supernatant (*R*^2^ > 0.996 for all curves). Parallelism was observed between the slopes of the calibrator and two batches of commercial rCL-11 (Figure 2A). Log-transformed values fitted a linear regression with *R*^2^ > 0.98 and the slopes (ranging from 0.56 to 0.58) did not differ significantly (*p* > 0.05). Parallelism was also observed between the slopes of serial dilutions of the calibrator and serum and plasma pools in the range 0.29–37.5 ng/ml, with slopes between −0.7 and −0.77 that were not significantly different (*p* > 0.05) (Figure 2B). The limit of detection, defined as the mean background absorbance plus two times the SD, was 0.17 and 0.19 ng/ml in serum and plasma respectively. The precision of the assay, evaluated using the ratio between individual OD values and the mean OD values of triplicate measurements for five different donors (Figure 2C), was found to be acceptable (CV < 20%) for concentrations above 0.29 ng/ml. To determine the dilution linearity the ratio between the interpolated concentrations and the mean concentration of a plasma pool was calculated for each dilution of a 10-point dilution curve (Figure 2D). The dilution linearity was satisfactory in the range 0.15–18.75 ng/ml with a deviation under 20% from the mean concentration. The working range of the assay—defined based on the parallelism, limit of detection, precision, and dilution linearity—was 0.29–18.75 ng/ml.

Intra- and inter-assay variation were calculated to estimate the variation of the assay. The intra-assay CV, calculated by measuring one sample forty times on the same plate, was 3.96 % (Table 1). The inter-assay CV was 10.5%, measured in triplicates on four plates analyzed on six different occasions.

**Table 1:** Intra- and inter-assay variation of ELISA.

	**Sample**	***n***	**CL-11(ng/ml)**		**SD**	**CV(%)**
			Mean	Range		
**INTRA-ASSAY VARIATION**						
	Plasma	40	282.6	250.2–299.1	11.2	3.96
**INTER-ASSAY VARIATION**						
	Plasma	6	286.7	257–335.2	30.1	10.5
	Serum	6	247.1	187.8–331.7	49.47	20.02

*The intra-assay variation was calculated by measuring a plasma pool sample on the same microtiter plate. The inter-assay variation was determined by comparing the mean of triplicates of a serum and plasma pool on four microtiter plates on six different days*.

### Effect of storage and freeze-thawing

**Figure 3:**
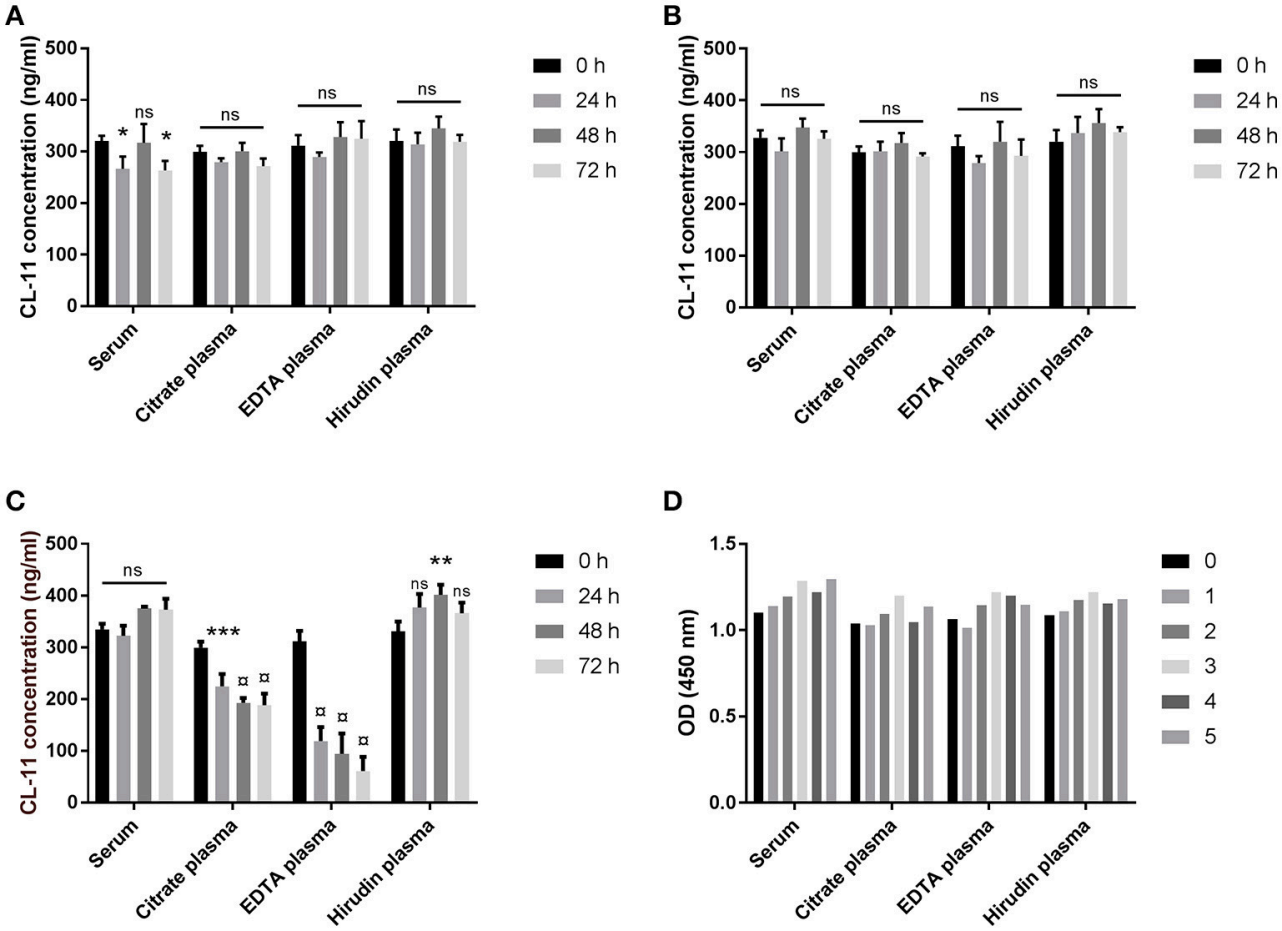
Sample stability. Effect of temperature on CL-11 concentration in serum and plasma from three donors after storage at 4°C **(A)**, RT **(B)**, or 37°C **(C)** for three days. **(D)** Effect of freeze-thawing cycles on CL-11 measurements serum and plasma from the same donor. *p < 0.05, **p < 0.01; ***p < 0.001; ¤, *p* = 0.0001.

The effect of temperature on the stability of CL-11 in blood samples was evaluated on matched serum and plasma from three different donors stored at 4°C, RT, or 37°C for up to three days, whereas the effect of freezing and thawing was studied on serum and plasma samples from a single donor over five consecutive cycles. No significant trend on CL-11 levels was observed when the samples were stored at 4°C (Figure 3A) or at RT (Figure 3B), nor after repeated freeze-thawing cycles (Figure 3D). On the other hand, storage at 37°C degrees caused a highly significant drop in CL-11 concentration in citrate and EDTA plasma (Figure 3C). CL-11 levels in serum and plasma did not differ significantly.

### CL-11 levels in plasma

**Figure 4:**
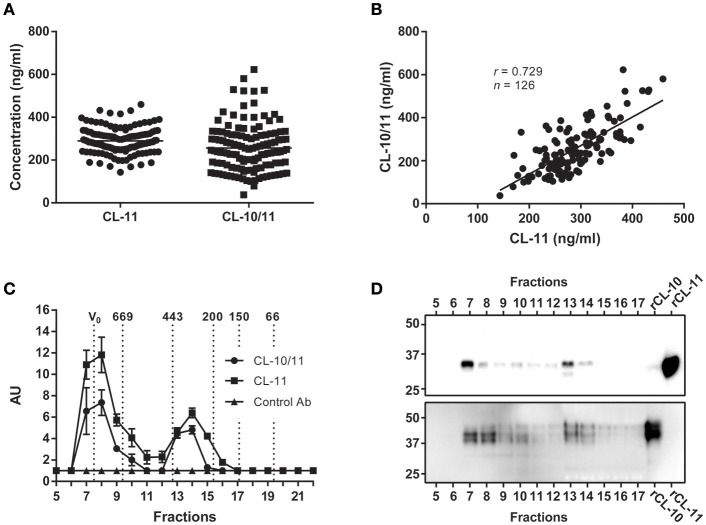
Concentration and molecular weight distribution of CL-11 and CL-10/11 complexes in plasma. **(A)** CL-11 and CL-10/11 concentration in plasma of 126 Danish blood donors using the validated assay described here and a semi quantitative CL-10/CL-11 sandwich ELISA. Horizontal lines represent mean. **(B)** Spearman's rank correlation between measured CL-11 and CL-10/11 levels. **(C)** Detection of CL-11 and CL-10/11 complexes in the elution fractions of size exclusion chromatography of plasma by sandwich ELISA. Relative levels of CL-11 and CL-10/11 complexes in the elution fractions are reported as arbitrary units (AU). Vertical dotted lines indicate the molecular masses of thyroglobulin (669 kDa), apoferritin (443 kDa), β-amylase (200 kDa), alcohol dehydrogenase (150 kDa), BSA (66 kDA), carbonic anhydrase (29 kDa). Western blot of elution fractions from size exclusion chromatography using Hyb-6 anti-CL-11 (**D above**) or anti-CL-10 (**D below**). rCL-11 and rCL-10 produced in CHO cells were used as positive controls.

CL-11 concentration in plasma was calculated from EDTA plasma samples of 126 Danish blood donors (mean 289.4 ng/ml, range 143.2–459.4 ng/ml) using rCL-11 as calibrator (Figure 4A). Since it has been reported that a major fraction of CL-11 exists in complex with the homologous CL-10, we measured the same samples by sandwich ELISA using an anti-CL-10 as capture antibody and anti-CL-11 Hyb-15 as detection. The estimated mean concentration of CL-10/11 complexes in plasma was 256.2 μg/ml (range 37.89–622.7) and there was a strong correlation between CL-11 and CL-10/11 levels (*r* = 0.729, Figure 4B. A pool of EDTA plasma from 10 healthy individuals was subjected to size exclusion chromatography followed by sandwich ELISA in order to define the relationship of CL-11 and CL-10/11 across their molecular weight distribution (Figure 4C). CL-11 and CL-10/11 migrated as two major oligomerization forms of approximately 400 and >600 kDa. ELISA results were confirmed by western blot using anti-CL-11 (Figure 4D above) and anti-CL-10 antibodies (Figure 4D below), where most of the protein is observed in fractions 7–8 and 13–14. Under reducing conditions, CL-10 migrates as a triple band of ~40 kDa. CL-11 present in the high molecular weight complexes migrates as a single band of ~34 kDa, whereas in the lower oligomers it appears as a double band—hinting at the occurrence of two different isoforms.

In light of the tight association between CL-11 and CL-10, we conclude that the ELISA developed in the current work is accurate and relevant to describe the natural situation of CL-11 in the circulation.

### CL-11 levels in patients with systemic inflammatory response syndrome (SIRS)

**Figure 5:**
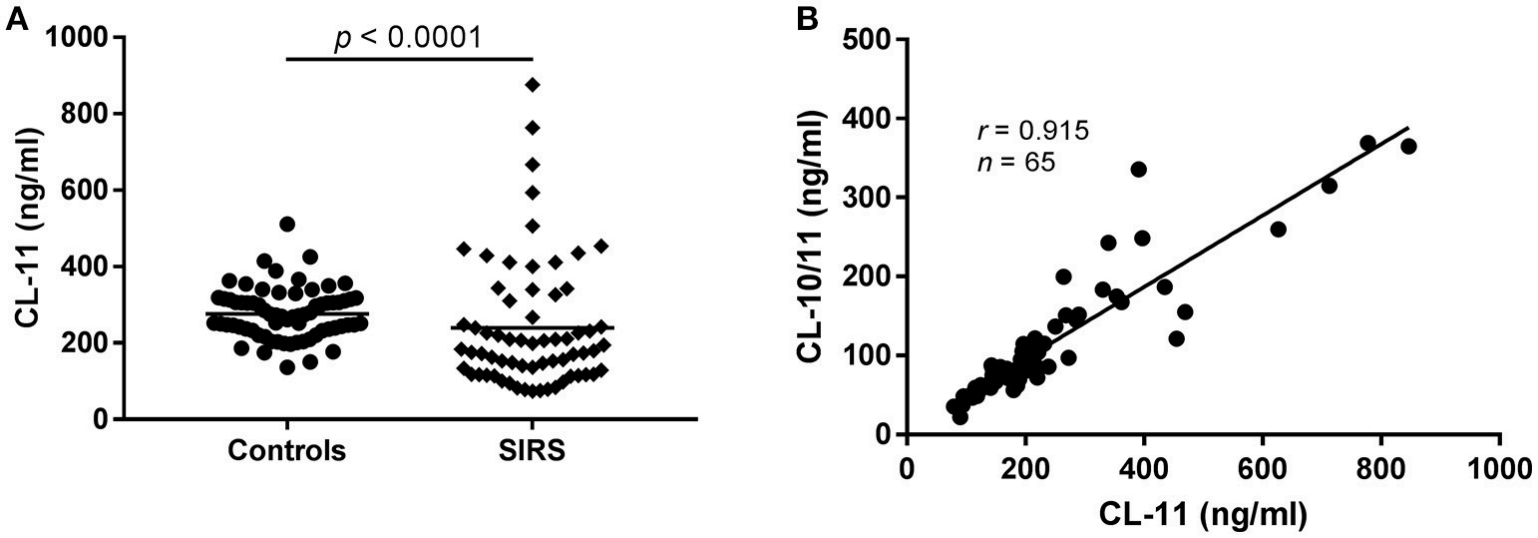
CL-11 levels in plasma from patients with SIRS and controls. **(A)** CL-11 concentration was measured in 65 SIRS plasma samples and 69 controls using the quantitative ELISA described here. Horizontal lines represent mean. **(B)** Spearman's rank correlation between CL-11 and CL-10/11 concentration in SIRS plasma samples.

Since CL-11 is expected to participate in the response against invading pathogens by activating the lectin pathway of complement, we measured the levels of CL-11 in 65 randomly selected plasma samples from patients admitted to the intensive care unit with SIRS [reported elsewhere(21)] (Figure 5A). Compared to healthy controls (*n* = 69), CL-11 was significantly decreased in patients with SIRS and spanned a wider range of concentrations (mean 276.4 ng/ml, range 136.2–511.7 vs. 240 ng/ml, range 73.76–876.4 ng/ml for controls and SIRS patients, respectively). As observed in healthy individuals, the concentration of CL-11 in SIRS plasma was strongly correlated with that of CL-10/11 complexes (*r* = 0.915, Figure 5B).

### Ligand characterization

**Figure 6:**
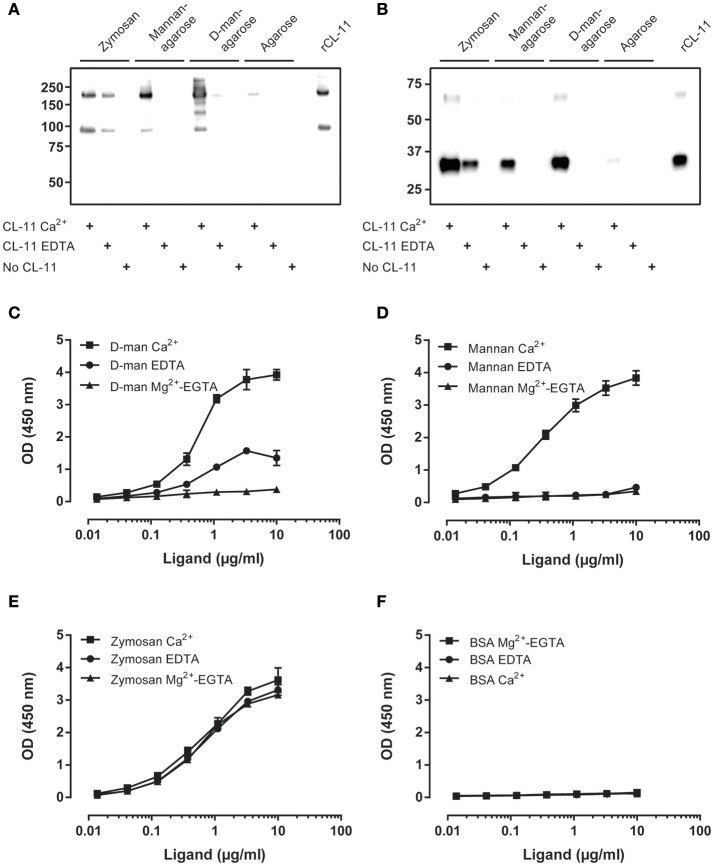
Characterization of CL-11 ligand binding. Western blot of rCL-11 co-precipitated with zymosan particles, mannan-agarose, D-mannose-agarose, or Sepharose in the presence of Ca^2+^ or EDTA under non-reducing **(A)** and reducing conditions **(B)**. Binding of rCL-11 (1 μg/ml) to serial dilutions of coated D-mannose-BSA **(C)**, mannan **(D)**, zymosan **(E)**, and BSA **(F)** in the presence of Ca^2+^, EDTA, and Mg^2+^-EGTA. Data is reported as mean ± SEM of three independent experiments.

Using our validated antibodies, we studied the ligand binding characteristics of CL-11 to identify a suitable ligand for *in vitro* complement activation assays. Binding was performed in the presence of calcium or EDTA (as cationic chelator) in the fluid phase using zymosan particles, mannan-agarose, D-mannose-agarose, and Sepharose as negative control. CL-11 displayed a characteristic C-type lectin activity and bound to D-mannose and mannan in the presence of calcium (1, 6) (Figures 6A,B). Remarkably, CL-11 was also able to bind to zymosan in a calcium-independent manner, suggesting an alternative ligand recognition mechanism. We confirmed the results by coating two-fold dilutions of mannan, zymosan, D-mannose-BSA, and BSA as negative control (Figures 6C–F). Binding of rCL-11 (1 μg/ml) was performed in the presence of calcium, EDTA, and magnesium-EGTA (a commonly used buffer for alternative pathway activation). As seen in the fluid-phase assay, calcium was required for binding to mannan and D-mannose (Figures 6C,E) but not to zymosan (Figure 6D). No binding to BSA was observed regardless of the buffer (Figure 6F).

**Figure 7:**
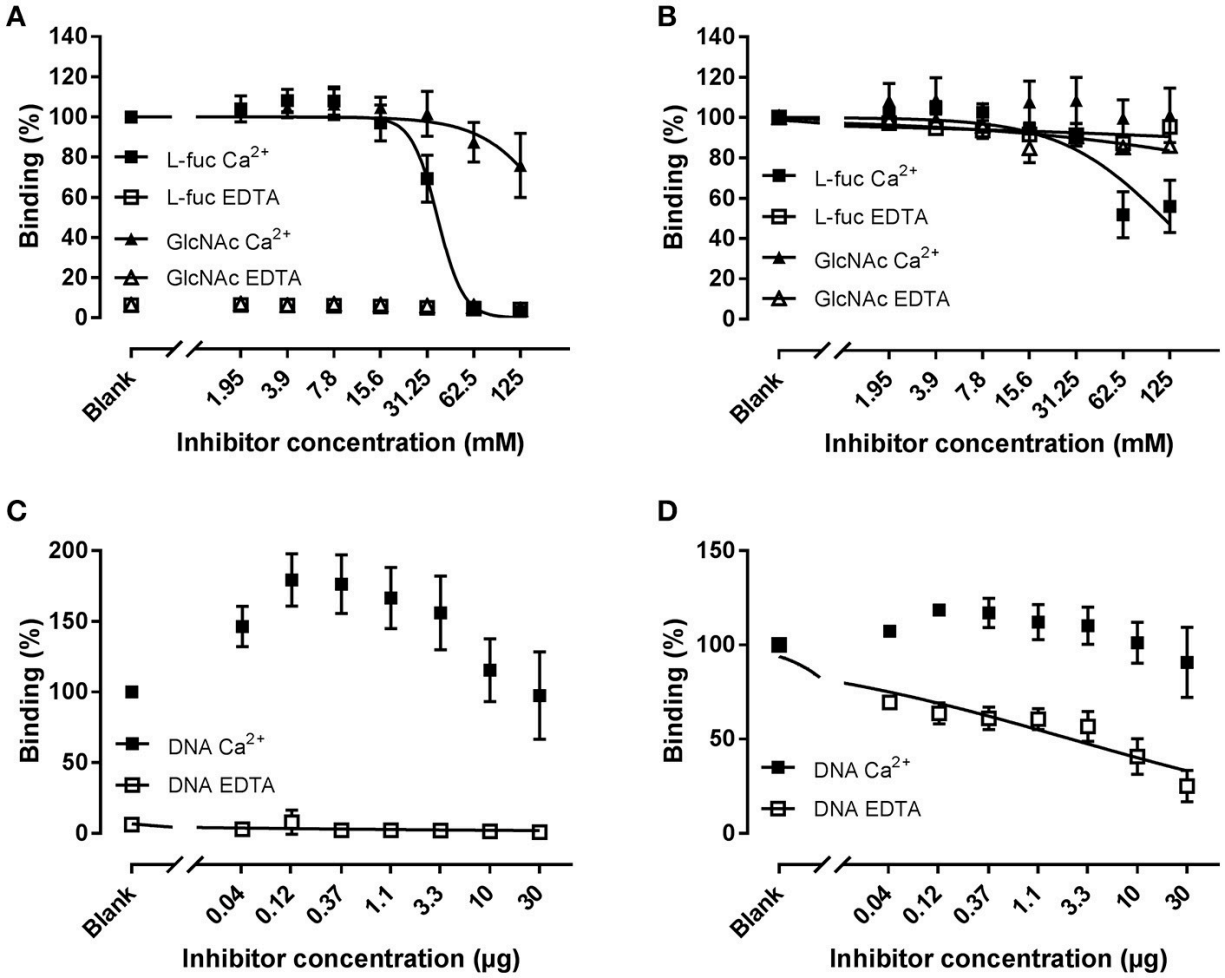
Inhibition of binding of rCL-11 to coated mannan **(A,C)** or zymosan **(B,D)**. Serial dilutions of L-fucose (L-fuc), N-acetyl-glucosamine (GlcNAc), or genomic DNA were incubated with rCL-11 (1 μg/ml) in the presence of Ca^2+^ or EDTA. Data is reported as percent binding compared to the well without inhibitor. Bound rCL-11 was measured with biotinylated mAb anti-CL-11 Hyb-15. Connecting lines represent nonlinear regression fits using the equation inhibitor concentration vs. response with variable slope. Data is reported as mean ± SEM of three independent experiments.

In order to determine whether the observed binding profiles are a result of the involvement of different binding sites for mannan and zymosan, we compared the effect of L-fucose (a strong ligand), N-Acetyl glucosamine (GlcNAc, a weak ligand), and genomic DNA (a calcium-independent ligand) on the binding to mannan and zymosan (Figure 7). Briefly, CL-11 was co-incubated with serial dilutions of L-fucose or GlcNAc starting in a 125 mM, or human genomic DNA starting at 30 μg/ml prior to addition to coated plates. L-fucose was an effective inhibitor of the binding to mannan in the presence of calcium with an IC50 of 36.48 mM, whereas N-acetyl-glucosamine had just a minor effect (IC50 > 125 mM) (Figure 7A). On zymosan, L-fucose was only a moderate inhibitor in the presence of calcium, forming an apparent plateau around 50% inhibition, while GlcNAc had no effect (Figure 7B). The limited effect of L-fucose suggests the presence of two separate binding sites for zymosan and carbohydrates.

CL-11 has been reported to bind to DNA in a calcium-independent fashion via an alternative binding-site. Co-incubation with DNA in the presence of calcium increased the binding to mannan, suggesting a cooperative effect between both molecules (see Discussion) (Figure 7C). DNA did not affect zymosan binding under calcium conditions, whereas in EDTA it showed a pronounced inhibition with an IC50 of 2.3 μg/ml (Figure 7D). These results indicate the presence of a shared binding site for nucleic acids and zymosan. Bearing all of the above in mind, we have identified a novel interaction with zymosan independent of divalent cations that we hypothesize may be relevant in environments where the binding to calcium is impaired, such as the gastrointestinal tract.

### CL-11-mediated C4 deposition on zymosan

**Figure 8:**
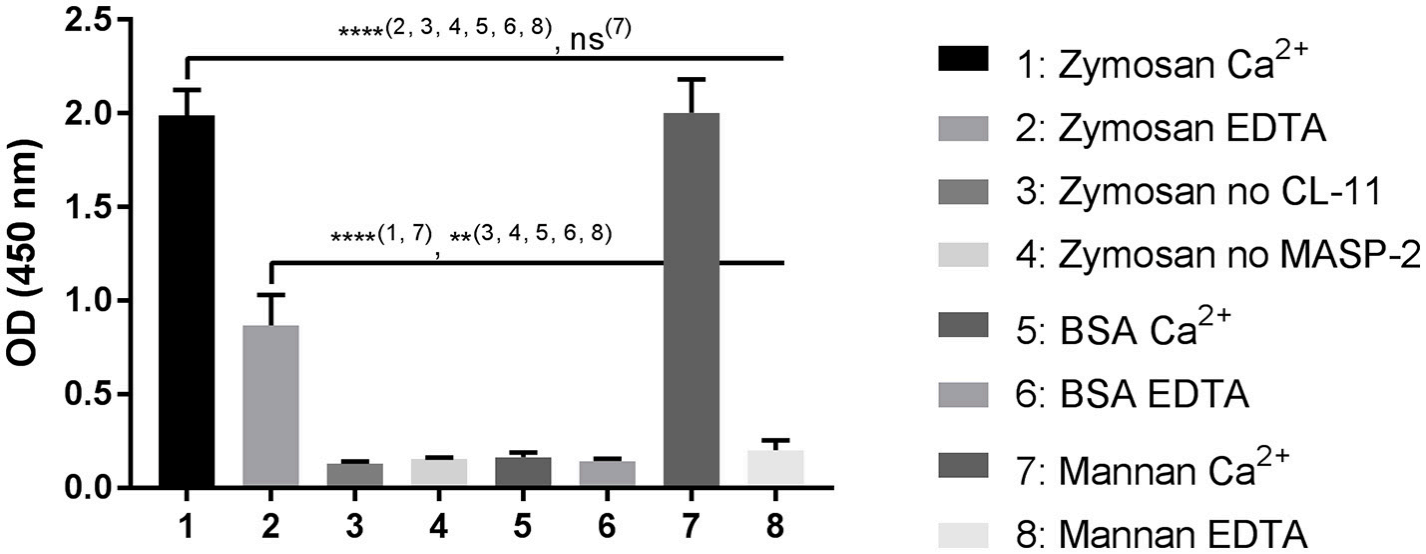
C4 deposition on zymosan. CL-11 (2 μg/ml) was allowed to bind to zymosan or mannan-coated plates (10 μ/ml) for 2 h in the presence of calcium or EDTA, followed by incubation with rMASP-2 (0.5 μg/ml) and purified C4 (0.5 μg/ml) for 4 h and 1 h respectively at 37°C. C4b deposition was measured using a pAb anti-C4c. Data is reported as mean ± SEM of three independent experiments. Numbers between parentheses are columns compared by ANOVA. **p < 0.01; ****p < 0.0001.

To investigate if CL-11 bound to zymosan could trigger the lectin complement pathway, we developed an *in vitro* assay using rMASP-2 and purified C4 (Figure 8). CL-11/MASP-2 complexes on zymosan caused a significant C4b deposition under calcium conditions in a level comparable to mannan. In the presence of EDTA, C4 activation was only seen on the zymosan ligand. No C4b deposition was observed on BSA, nor in the absence of CL-11 and MASP-2.

## Discussion

We have generated mAbs against human CL-11 and established a quantitative sandwich ELISA. The antibody-producing hybridomas underwent successive selection rounds using first rCL-11, and later serum and plasma by ELISA and western immunoblotting. While all clones readily bound rCL-11, just a few reacted strongly with native CL-11. This is probably due to the existence of complexes in the circulation that hide epitopes otherwise exposed in the homomeric rCL-11 used as immunogen. In fact, immunoprecipitation of serum demonstrates that native CL-11 has a different oligomeric distribution than our recombinant protein (Figure 1). The best candidates were pairwise compared to develop a sandwich ELISA. Two antibodies were selected as capture and detection antibody in the basis of their signal-to-noise ratio. The assay was validated in terms of linearity, variability, and precision, demonstrating a satisfactory variability, and parallelism between calibrator and serum and plasma across a working range of 0.29–18.75 ng/ml (Figure 2). We observed no significant differences between CL-11 concentration in serum and four different widely used plasma preparations (Figure 3). Similarly, common storage conditions and freeze-thawing procedures did not affect the measurements of CL-11, highlighting the suitability of this assay for routine measurements in the clinic. Of interest, some of the mAbs cross-reacted with CL-11 from other species, granting the possibility of forthcoming studies into CL-11 in animal models of disease.

When optimizing the sandwich ELISA we noticed that EDTA was required to detect native CL-11 in serum and plasma (but not rCL-11) (Supplementary Figures 1A–C). Moreover, Western blot results following immunoprecipitation of serum CL-11 in a calcium-containing buffer vs. EDTA buffer resulted in a different banding pattern (Supplementary Figure 1D). Calcium stabilizes the tertiary structure of the CRD of CL-11 (10), and is required for the association of the MASPs with the collagen stalks of the PRMs (22, 23). To determine whether the EDTA-dependent detection of native CL-11 was due to a change in its three-dimensional structure or masking of the epitope by bound MASPs, we incubated rCL-11 in the presence of calcium, EDTA, and MASP-3. Calcium and MASP-3 had no effect on the detection of rCL-11. It has been frequently observed that serum can inhibit the binding of spiked collectins and ficolins to their ligands (14). This inhibition is greatly reduced when using EDTA in the dilution buffer. At the same time others have reported the use of a calcium-dependent antibody for the purification of native CL-10/11 complexes (24). Our hypothesis is that CL-11 (or CL-10) exists in a calcium-dependent complex with a hitherto unknown protein that both interferes with detection of native CL-10/11 complexes at low serum dilutions and inhibits the binding of spiked CL-11.

**Figure 9:**
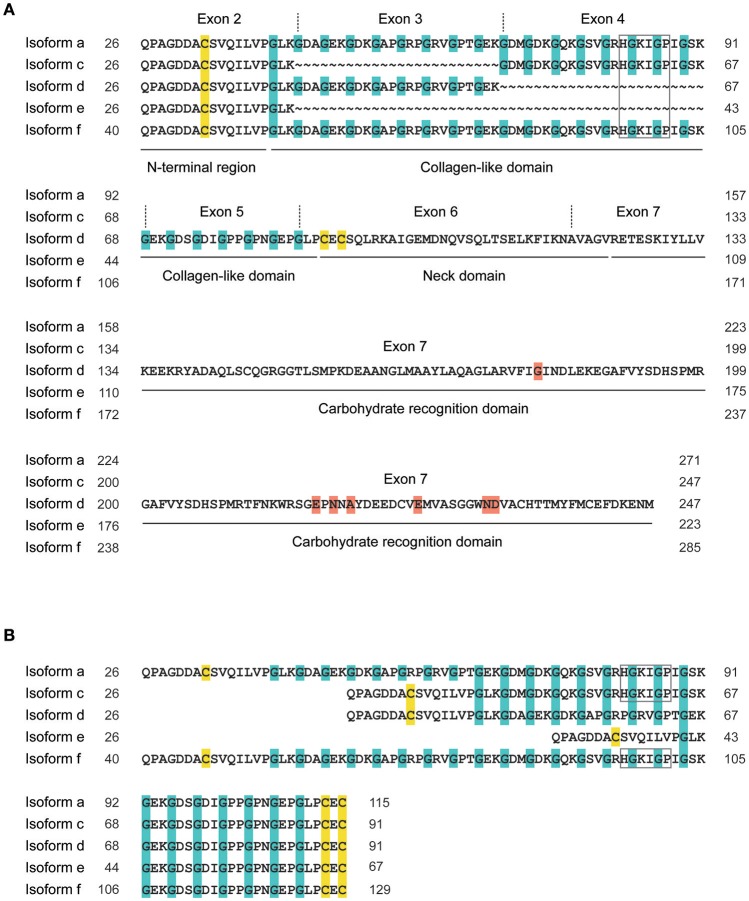
Exon and domain organization of CL-11 isoforms. The five isoforms predicted to be secreted are aligned from the N-terminus **(A)** with the missing amino acids represented by tildes (~), or from the cysteine pair in the neck domain **(B)**. Exons 5 to 7, numbered according to isoform a, are shown once for they are conserved between isoforms. Cysteines are highlighted in yellow, glycines in Gly-X-Y collagen repeats in blue, amino acids involved in carbohydrate binding in red. Grey box, putative MASP-binding motif. Isoform a, NM_024027.4; isoform c, NM_001255982.1; isoform d, NM_001255983; isoform e, NM_001255984.1; isoform f, NM_001255985.1.

Since it appears that CL-11 is found in the circulation associated with the homologous CL-10 (14), we used our assay and a semiquantitative ELISA to compare the levels of CL-11 and CL-10/11 complexes in plasma from Danish blood donors (Figure 4). The mean plasma concentration of CL-11 was 289.4 ng/ml, range 143.2–459.4 ng/ml, in agreement with two previously published Danish and Japanese cohorts (284 ng/ml and 340 ng/ml, respectively) (7, 25), and equivalent to the estimated concentration of CL-10/11 (256.2 ng/ml, range 37.89–622.7 ng/ml). However, we want to remark that lacking a carefully validated ELISA for CL-10/11, the later concentration is merely an educated guess. There was a strong correlation between CL-11 and CL-10/11 levels in plasma (*r* = 0.729) apparent across a wide range of molecular masses after subjecting plasma to size exclusion chromatography and measuring the levels of CL-11 and CL-10/11 in the elution fractions by ELISA and western blot. This tight association previously reported for CL-11 and CL-10 (26–28), conforms with quantitative mass spectrometry estimates suggesting that the CL-10/11 subunit is composed of one CL-11 and two CL-10 polypeptide chains (14). Plasma CL-11 and CL-10/11 eluted as two distinct peaks at 300–400 kDa and >600 kDa that may correspond with tetramers of trimeric subunits and higher forms, respectively. Although direct area-under-the-curve comparisons suggest that most of CL-10 and CL-11 exist as high molecular weight complexes in the circulation, the lack of resolution beyond 700 kDa makes it impossible to differentiate hexamers of trimers from higher oligomeric forms, that could otherwise elute in distinct peaks with a similar area-under-the-curve as the ~400 kDa peak. Western blot of the elution fractions under-reducing conditions revealed multiple monomer bands for CL-10 and CL-11 in agreement with the observations of Henriksen et al. that CL-10 is found in the circulation as three glycosylated states and CL-11 as two differentially-spliced isoforms (14). Detection of CL-11 demonstrated the presence of a second band in the low molecular weight complexes, possibly corresponding to the shorter isoform D. Of the five isoforms predicted to be secreted by *in silico* analyses (29), only isoform A and D have been detected in blood (1, 14, 25). Isoform D has a shorter collagen-like domain (48 amino acids as opposed to 72 in isoform A) and lacks the putative MASP-binding motif HGKIGP (Figure 9). Though shorter, isoform D maintains intact the Gly-X-Y repeats of the collagen-like domain required for the formation of interchain hydrogen bonds and stabilization of the collagen triple helix (30, 31). Because the C-terminus of isoforms A and D are identical, we hypothesize that the neck domain could direct the assembly of a trimer with a ragged N-terminal region with cysteines incapable of stabilizing high order oligomeric structures (32). Detection of CL-10 in the elution fractions revealed that non- or singly-glycosylated CL-10 was more abundant in the ~600 kDa oligomers, while the doubly-glycosylated form was more abundant in the ~400 kDa complexes. It has been suggested that CL-11 stabilizes and facilitates secretion of CL-10 (14), in part due to the difficulties of expressing the CL-10 recombinantly (33, 34). In the current work we describe the production of rCL-10 using stable transfected CHO cells. The protein was secreted into the supernatant and appeared to form oligomers of trimers (data not shown). Further studies in the characterization of homomeric CL-10 and the influence of alternative spliced variants and glycosylation states in the oligomerization of CL-10/11 are guaranteed, as are studies on the effect (if any) of the isoform D lacking the MASP binding motif in the weak interaction of low molecular weight CL-10/11 with the MASPs.

Based on the putative role of CL-11 in the modulation of the immune response against pathogens (2, 9), we performed a pilot study on randomly-selected plasma samples from patients diagnosed with SIRS (Figure 5). Notably, the mean CL-11 plasma concentration was significantly decreased in SIRS patients upon admission compared to healthy controls (*p* < 0.0001). Nonetheless, we choose to remain cautious and refrain from any biological interpretation until a thorough analysis of new ongoing projects can be performed. The levels of homomeric and heteromeric CL-11 were strongly associated in SIRS patients (*r* = 0.915), as seen for healthy controls, reinforcing the current opinion that CL-10 and CL-11 are co-regulated and exist as a hybrid molecule.

CL-11 has been shown to bind to several microorganisms (6), with a preference for complex sugars as opposed to other collectins (10). Using the developed mAbs we confirmed that our in-house recombinant protein displays the characteristics of native CL-11 and binds to mannan and D-mannose in a calcium-dependent manner (Figure 6). As expected, L-fucose—but not GlcNAc—was an effective inhibitor of the binding to mannan (IC50 = 36.48 mM). Interestingly, rCL-11 also bound to zymosan in a calcium-independent manner that could be only partially inhibited by L-fucose in the presence of calcium. Zymosan is a major constituent of the cell wall of the yeast *Saccharomyces cerevisiae*. It is composed mainly of cross-linked β-glucan and α-mannan, and has been widely used to elicit immune and inflammatory responses (35–38). Several calcium-independent ligands, such as nucleic acids, have been reported for CL-11 reflecting complex interactions beyond its lectin activity (2, 10, 19). Thus, we incubated rCL-11 with DNA and measured the amount of bound CL-11 to coated mannan and zymosan (Figure 7). On mannan DNA appeared to increase the binding to the coated ligand. The same phenomenon has been reported by Henriksen et al., where the authors concluded that DNA may increase the avidity or align the CRDs in a more favorable conformation (19). On zymosan, DNA had a minor effect in calcium, while in EDTA buffer it was a moderately effective inhibitor (IC50 = 2.3 μg/ml). We propose that CL-11 recognizes zymosan via two distinct mechanisms: a classical calcium-dependent lectin activity, and an alternative calcium-independent interaction shared with nucleic acids. Other C-type lectins, such as dectin-1 and Reg family proteins III and IV, are also capable of recognizing fungal and bacterial ligands in a metal ion-independent fashion (39–41). Interestingly, it has been proposed that the Reg family proteins evolved to bind sugars without calcium to carry their antimicrobial function in the gastrointestinal tract, where the low pH may impair the ability of classical C-type lectins to bind to carbohydrates. Similar low pH, low calcium conditions result from infection-induced local inflammation (42). Immunohistochemical analyses have revealed that CL-11 is expressed (among others) in the stomach and the intestines (11). At this stage we can only conjecture whether the calcium-independent zymosan interaction may be of biological relevance in acidic environments with low calcium availability.

Finally, we measured the ability of rCL-11 to trigger lectin pathway activation on zymosan (Figure 8). It has been reported that serum inhibits the binding of purified and recombinant collectins and ficolins to their ligands (14). At the same time, the relatively low concentration of CL-11 in circulation requires using serum dilutions that would trigger complement activation via the alternative pathway. To circumvent these limitations, we employed a step-wise activation assay using recombinant and purified proteins in a buffer that allows the cleavage of C4 by MASP-2—the first step of the activation of the lectin pathway. Binding of rCL-11 followed by incubation with rMASP-2 and purified C4 resulted in the deposition of C4b on coated zymosan, comparable to that on mannan. Calcium was not necessary for the binding of rCL-11 to zymosan, but for the complex formation with rMASP-2. We consider that not requiring calcium is an asset when designing a specific assay to discriminate the contribution of CL-11 from the other PRMs of the lectin pathway.

In conclusion, we have developed monoclonal antibodies and a robust and sensitive sandwich ELISA for the quantification of CL-11 in the circulation and used them to study native CL-11 in plasma and the binding characteristics of rCL-11 to different ligands.

## Author contributions

RB-O, NK-M, LP-A, and KS performed the experiments. RB-O, KS, M-OS, and PG designed the study. RB-O, PG wrote the manuscript. All authors critically reviewed the manuscript.

### Conflict of interest statement

The authors declare that the research was conducted in the absence of any commercial or financial relationships that could be construed as a potential conflict of interest.

## Supplementary material

Supplementary Figure 1 Effect of calcium in the detection of native CL-11. The concentration of CL-11 was measured in three different donors in a three-fold dilution of serum **(A)** and plasma **(B)** or supernatant from CHO cells **(C)** with and without EDTA the sample buffer. **(D)** Western blot of immunoprecipitation (IP) of CL-11 from serum in a calcium or EDTA-containing buffer under non-reducing conditions. Lanes 1 and 2, purified rCL-11 and supernatant from CHO cells expressing rCL-11. Lane 3, serum before IP. Lanes 4 to 9, serum after IP using CL-11 specific mAbs Hyb-15 and Hyb-17 or a mouse IgG1κ isotype control antibody. The blots were developed with pAb rabbit anti-CL-11. ELISA, IP and Western blotting were performed according to sections CL-11 Specific Sandwich ELISA, Immunoprecipitation, SDS-page and western blot, respectively.
