# Supplementary figures and images for: Development of a Quantitative Assay for the Characterization of Human Collectin-11 (CL-11, CL-K1)
Source: Front Immunol. 2018 Sep 28;9:2238. doi: 10.3389/fimmu.2018.02238 (PMC6172411; doi:10.3389/fimmu.2018.02238)

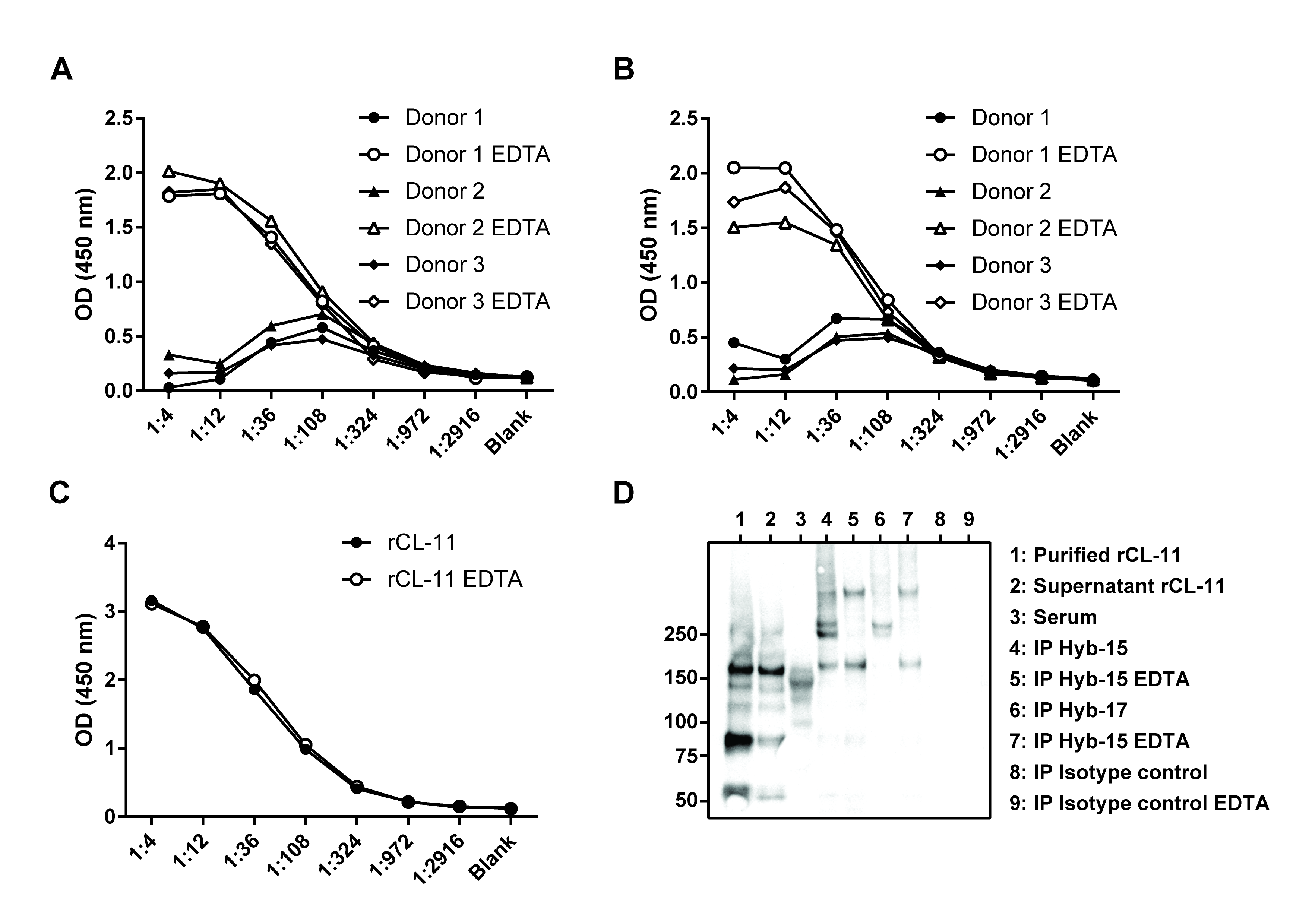

Supplement: Supplementary Figure 1 — Effect of calcium in the detection of native CL-11. The concentration of CL-11 was measured in three different donors in a three-fold dilution of serum (A) and plasma (B) or supernatant from CHO cells (C) with and without EDTA the sample buffer. (D) Western blot of immunoprecipitation (IP) of CL-11 from serum in a calcium or EDTA-containing buffer under non-reducing conditions. Lanes 1 and 2, purified rCL-11 and supernatant from CHO cells expressing rCL-11. Lane 3, serum before IP. Lanes 4 to 9, serum after IP using CL-11 specific mAbs Hyb-15 and Hyb-17 or a mouse IgG1κ isotype control antibody. The blots were developed with pAb rabbit anti-CL-11. ELISA, IP and Western blotting were performed according to sections CL-11 Specific Sandwich ELISA, Immunoprecipitation, SDS-page and western blot, respectively. [file Image_1.TIF]
